# Achievable agricultural soil carbon sequestration across Europe from country‐specific estimates

**DOI:** 10.1111/gcb.15897

**Published:** 2021-10-06

**Authors:** Leonor Rodrigues, Brieuc Hardy, Bruno Huyghebeart, Julia Fohrafellner, Dario Fornara, Gabriela Barančíková, Teresa G. Bárcena, Maarten De Boever, Claudia Di Bene, Dalia Feizienė, Thomas Kätterer, Peter Laszlo, Lilian O’Sullivan, Daria Seitz, Jens Leifeld

**Affiliations:** ^1^ Climate and Agriculture Group Agroscope Reckenholz Zürich Switzerland; ^2^ Walloon Agricultural Research Centre Gembloux Belgium; ^3^ BIOS Science Austria Vienna Austria; ^4^ Agri‐Food & Biosciences Institute (AFBI) Belfast UK; ^5^ Faculty of Management Prešov University Prešov Slovakia; ^6^ National Agricultural and Food Centre Lužianky Slovakia; ^7^ Norwegian Institute of Bioeconomy Research (NIBIO) Ås Norway; ^8^ Plant Sciences Unit Flanders Research Institute for Agriculture Fisheries and Food (ILVO) Merelbeke Belgium; ^9^ CREA ‐ Council for Agricultural Research and Economics Research Center for Agriculture and Environment Rome Italy; ^10^ Institute of Agriculture Lithuania Research Centre for Agriculture and Forestry Akademija Lithuania; ^11^ Department of Ecology Swedish University of Agricultural Sciences Uppsala Sweden; ^12^ ATK ‐ Institute for Soil Sciences Centre for Agricultural Research Budapest Hungary; ^13^ Teagasc, Environment, Soils & Land Use Department Johnstown Castle Co Wexford Ireland; ^14^ Thünen‐Institute of Climate‐Smart Agriculture Braunschweig Germany

**Keywords:** 4 per 1000 initiative, agricultural management, climate change, Europe, GHG mitigation, soil carbon sequestration

## Abstract

The role of soils in the global carbon cycle and in reducing GHG emissions from agriculture has been increasingly acknowledged. The ‘4 per 1000’ (4p1000) initiative has become a prominent action plan for climate change mitigation and achieve food security through an annual increase in soil organic carbon (SOC) stocks by 0.4%, (i.e. 4‰ per year). However, the feasibility of the 4p1000 scenario and, more generally, the capacity of individual countries to implement soil carbon sequestration (SCS) measures remain highly uncertain. Here, we evaluated country‐specific SCS potentials of agricultural land for 24 countries in Europe. Based on a detailed survey of available literature, we estimate that between 0.1% and 27% of the agricultural greenhouse gas (GHG) emissions can potentially be compensated by SCS annually within the next decades. Measures varied widely across countries, indicating differences in country‐specific environmental conditions and agricultural practices. None of the countries' SCS potential reached the aspirational goal of the 4p1000 initiative, suggesting that in order to achieve this goal, a wider range of measures and implementation pathways need to be explored. Yet, SCS potentials exceeded those from previous pan‐European modelling scenarios, underpinning the general need to include national/regional knowledge and expertise to improve estimates of SCS potentials. The complexity of the chosen SCS measurement approaches between countries ranked from tier 1 to tier 3 and included the effect of different controlling factors, suggesting that methodological improvements and standardization of SCS accounting are urgently required. Standardization should include the assessment of key controlling factors such as realistic areas, technical and practical feasibility, trade‐offs with other GHG and climate change. Our analysis suggests that country‐specific knowledge and SCS estimates together with improved data sharing and harmonization are crucial to better quantify the role of soils in offsetting anthropogenic GHG emissions at global level.

## Graphical abstract

Country‐specific soil carbon sequestration (SCS) potentials, of agricultural land of mineral soils, for 24 countries in Europe are evaluated. Available knowledge on national achievable SCS potentials is still limited, and only half of the analysed countries have explored nationwide SCS potentials. Information provided often does not consider practical and socio‐economic implications, which are vital for sustainable implementation. The SCS potentials do, however, point towards important contributions to mitigate climate change, covering considerable shares of national greenhouse gas emissions from the agricultural sector ranging from 0.1% to 27% annually. Data heterogeneity suggests that methodological improvements and standardization of SCS accounting are urgently required.

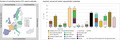

## 1 INTRODUCTION

To meet the Paris Agreement goal of limiting global average temperatures to below 2℃, preferably to 1.5℃, compared to pre‐industrial levels (UNFCCC, 2015), the EU aims to reduce 40% of its domestic greenhouse gas (GHG) emissions by 2030 and become the world's first climate‐neutral economy by 2050 (‘Green Deal’) (EC, 2019). Today, 11% of total European GHG emissions derive from agriculture and measures to reduce and offset these emissions are urgently required to meet climate mitigation targets (EU NIR, 2021). The role of soils in the global carbon cycle and the importance of reducing GHG emissions from agriculture has been increasingly acknowledged (IPCC, 2018). Depending on land use and management, soils can act either as source or sink of CO_2_ (Lal, 2004). Soils are the largest terrestrial pool of organic carbon, with global soil organic carbon (SOC) stocks estimated as 863, 1824 and 3012 Pg C in the upper 0.3, 1 and 2 m of soil respectively (Sanderman et al., 2017). Evidence from various long‐term soil‐monitoring and field experiments (FEs) across several European countries shows that organic carbon contents are decreasing in many agricultural soils (Bellamy et al., 2005; Heikkinen et al., 2013; Keel et al., 2019; Sleutel et al., 2007; Taghizadeh‐Toosi et al., 2014). However, there is general agreement that soil C losses can be reverted and C accumulation increased under selected agricultural practices, which could further benefit soil fertility and ecosystem service delivery (e.g. water infiltration and holding capacity, provision of food and ecological resilience, preventing soil erosion etc.) (Baveye et al., 2020; Lal, 2018). Hence, in the last two decades, more research has been focused on what certain agricultural practices (e.g. cover crops [CC], residue management, land use changes), might contribute to increase SOC stocks and help removing CO_2_ from the atmosphere as effective climate mitigation measures (Bolinder et al., 2020; Lal, 2004; Paustian et al., 2016; Smith et al., 2005, 2007).

The ‘4 per 1000’ (4p1000) initiative has become a prominent soil‐based climate mitigation action also to ensure food security through an annual increase in SOC stocks by 0.4%, or 4‰ per year, in the top 0–40 cm of soil. It was launched in 2015 at the United Nations Climate Change Conference (COP 21, CMP 11) together with the Paris agreement. Since then, the feasibility of the 4p1000 soil C sequestration annual target has been intensively debated. For example Rumpel et al. (2020) summarized the opportunities and limitations of soil carbon sequestration (SCS) as a sustainable development strategy and the implementation challenges of the 4p1000 initiative and Amelung et al. (2020) stressed the aspirational nature of the sequestration target.

Specific criticism of the 4p1000 initiative relates to the role of biophysical barriers (Baveye et al., 2018; Poulton et al., 2018; Schiefer et al., 2018; de Vries, 2018; White et al., 2018), trade‐offs (Lugato et al., 2018), climate change effects (Mondini et al., 2012) and socio‐economic and political implications (Baveye et al., 2018; Poulton et al., 2018; White et al., 2018), which all together can greatly affect the 4p1000 SCS target. Besides these well‐justified critiques and existing knowledge gaps, soil scientists generally agree that enhancing SCS comes with multiple benefits as associated with increased soil quality and greater resilience of soil ecosystems to human management. In this context, according to Olson et al. (2014) and Chenu et al. (2019), SCS is defined as ‘the process of transferring CO_2_ from the atmosphere into the soil of a land unit, through plants, plant residues and other organic solids which are stored or retained in the unit as part of the soil organic matter. The sequestered SOC should increase the net SOC storage during and at the end of a study to above the previous pre‐treatment baseline’. However, there is still much uncertainty about the extent to which SCS can be enhanced under different management practices, especially in relation to region‐specific potentials (Amelung et al., 2020).

To realistically estimate achievable and implementable SCS, the first step is to determine where and to which extent particular SCS measures can be mobilized. Furthermore, costs and practical applicability of these measures should be considered. The potential of SCS of agricultural land is generally estimated from modelling outputs calibrated with long‐term experiment (LTE) data. The first estimates of technical/biophysical agricultural SCS potentials for Europe were given by Smith et al. (1998), based on results from several LTEs. Under the assumption that 100% of the total arable land of Europe (EU15) is converted to no‐tillage (NT), Smith et al. (1998) estimated SCS potentials of 1.2–2.3 Gt C after 50 and 100 years respectively. A more realistic update combining SCS estimates from other LTEs across Europe was given by Freibauer et al. (2004). The study took into account the available area of agricultural land and its suitability for implementing selected management options and showed that annually 16–19 Mt C could be sequestered in arable soil of EU15. More recent estimates at the pan‐European (EU 28+Serbia, Bosnia and Herzegovina, Montenegro, Albania, Former Yugoslav Republic of Macedonia and Norway) level were given by Lugato et al. (2014) using the CENTURY agroecosystem model for a set of management practices under different land use scenarios. The study estimated that the implementation of different management practices (e.g. CC, conversion to grassland [GR], reduced tillage [RT]) on 12%–28% of European arable lands (EU28) would sequester from 27 to 91 Mt C by 2020 and from 150 to 583 Mt C by 2100 in the upper 30 cm of soil. This means that annual SCS potentials might range initially between 2.7 and 9.1 Mt C (Lugato, Bampa, et al., 2014), thus offsetting 2.3%–7.8% of total European emissions from the agricultural sector (excluding land use and land use change and forestry [LULUCF] and fossil energy sources used for agriculture).

These results underpin the significant role that agricultural soils can play in mitigating GHG emissions at the European scale. To be realistic, SCS scenarios must be designed at large scale (national or even regional level) and take into account local pedo‐climatic, socio‐economic and political environment, which are key for a sustainable and successful implementation. This is best achieved via estimates acquired from within countries, based on the premise that a more detailed knowledge on the individual applicability of SCS measures is available at national rather than international levels. Yet, country‐specific knowledge and estimates on SCS potentials are hitherto insufficiently exploited.

Here we present a reality check on where 24 European countries stand in relation to European GHG reductions targets through application of SCS management options and the 4p1000 initiative (knowledge, feasibility) using a bottom‐up approach by exploiting country‐specific knowledge and data sets. We give an overview of estimates of national SCS potentials for mineral agricultural soils related to a change in farming practices, the share of land for which such information is available and calculation methodology. The objectives of this study are to assess (1) the potential abatement of GHG emissions relative to GHG emissions of the agricultural sector through the implementation of country‐specific SCS measures; (2) the feasibility of the 4p1000 initiative for each country; and (3) major knowledge gaps associated with the estimation of SCS potential across multiple countries.

## 2 METHODS

This study was conducted within the framework of the Horizon 2020 European Joint Programme SOIL (EJP‐SOIL; https://ejpsoil.eu/). We specifically carried out a stocktake of estimates of achievable SCS of agricultural mineral soils across Europe based on an extensive review of available literature at national level. Country experts (*n* = 35) on soil C sequestration research were identified during the early steps of the stocktake exercise by contacting the EJP‐SOIL coordinators from each partner country. Available information was gathered at national level from both peer review literature, government reports and information from GHG inventories. In total, 24 countries (AT, BE, CH, CZ, DE, DK, EE, ES, FI, FR, HU, IR, IT, LT, LV, NL, NO, PO, PT, SI, SK, SW, TR and UK) were included in the study.

The stocktake aimed to collect state‐of‐the‐art knowledge on achievable SCS in agricultural mineral soils at national level. We specifically gathered data of quantitative estimations of achievable SCS under different measures including soil management practices, land use and land use changes across 24 countries.

Information from quantitative studies was collected and included as follows: (a) title of the study, (b) the form and accessibility of information (i.e. published scientific or commercial literature, grey literature etc.), (c) the type of measures involved and their SCS potentials, (d) the spatial scale on which the studies operated (regional, national), (e) the methods used to estimate or calculate the achievable SCS and (f) temporal scale for which SCS has been quantified.

### 2.1 Assessment of literature

Studies and articles were screened and included in the stocktake based on the following criteria: (a) *Subject relevance*: the stocktake was limited to studies reporting estimates of achievable SCS potentials of mineral (organic soils were excluded) cropland and GR soils (forest soils were excluded), under a specific change in land management (including land use changes). (b) *Relevant study design*: SCS assessment should meet the definition of Olson et al. (2014), namely that ‘the sequestered SOC should increase the net SOC storage during and at the end of a study to above the previous pre‐treatment baseline’. (c) *Relevant results*: SCS (e.g. Tg C year^−1^ of Tg CO_2‐eq_ year^−1^) must be expressed regarding a specific spatial scale (ha). To meet this goal, literature was first sorted by title and abstract and second by full text screening. All relevant studies were included in a meta‐database describing the reference, study settings, measures involved, temporal and spatial scale, methods and quantitative data (available in supporting information).

### 2.2 Analysis of articles

The studies were analysed according to:

- the methodology used to determine SCS potentials, sorted by level of complexity (Tiers 1–3 according to IPCC)
- parameters involved in the estimate of SCS (reasoning behind the determination of area on which measures are implemented [realistic area], technical and practical feasibility, trade‐offs with other GHG, climate change). In this study, we refer to *technical feasibility* when measures used by the studies are relevant to the country farming specificities. This includes measures which are already applied or the readiness of a technology for its implementation is given (including farmers knowledge, equipment, extension services) (Smith et al., 2008). To *practical feasibility*, we refer to the consideration of factors such as implementation costs, local policies, regulations and assessment of willingness of farmers.
- the value of potentials in relation to domestic emissions from the agricultural sector (excl. LULUCF), henceforth referred to as EA (i.e. Emissions from the agricultural sector). The EA was taken from the national GHG inventory reports of each country submitted in 2020 (National Inventory Submissions 2020|UNFCCC).
- the threshold value of the 4p1000 initiative. Accordingly, quantification of carbon stocks for a given soil depth under a given area and management type is a prerequisite to estimate the increase in soil carbon. For the sake of consistency (i.e. same method, soil depth), we used the soil carbon stocks calculated by Lugato, Panagos, et al. (2014) through the CENTURY agroecosystem model. These model outputs provide the baseline of soil carbon stocks to a depth of 30 cm (2010) for the total agricultural area (organic and mineral soils) excluding forests across Europe for our study.

The SCS potentials of the individual countries are further compared to modelled estimates made by Lugato, Bampa, et al. (2014), who estimated the annual SCS technical potentials of six individual measures (conversion from arable land to GR [AR‐GR], residue management [RES], ley arming [LEY], crop residue+reduced tillage [RET] and RT) from modelling with CENTURY, for different time horizons. The study further investigates three simple policy‐oriented scenarios where these measures are combined and applied to different shares of arable land 12% (S1), 24% (S2) and 28% (S3) equally distributed among all arable land (no geographic specification). S1 was characterized by the equal conversion of 12% of arable land to the six alternative practices. The S2 involved 24% of arable land with differing proportions of the six measures (5% AR‐GR, 5% RES, 5% RET, 5% RT, 2% LEY and 2% CC). The third scenario S3 involves 28% of arable with 10% AR‐GR, 2% RES, 2% RET, 2% RT, 5% LEY and 7% CC respectively. The three scenarios are compared to the national SCS potentials found in this study.

## 3 RESULTS

**FIGURE 1:**
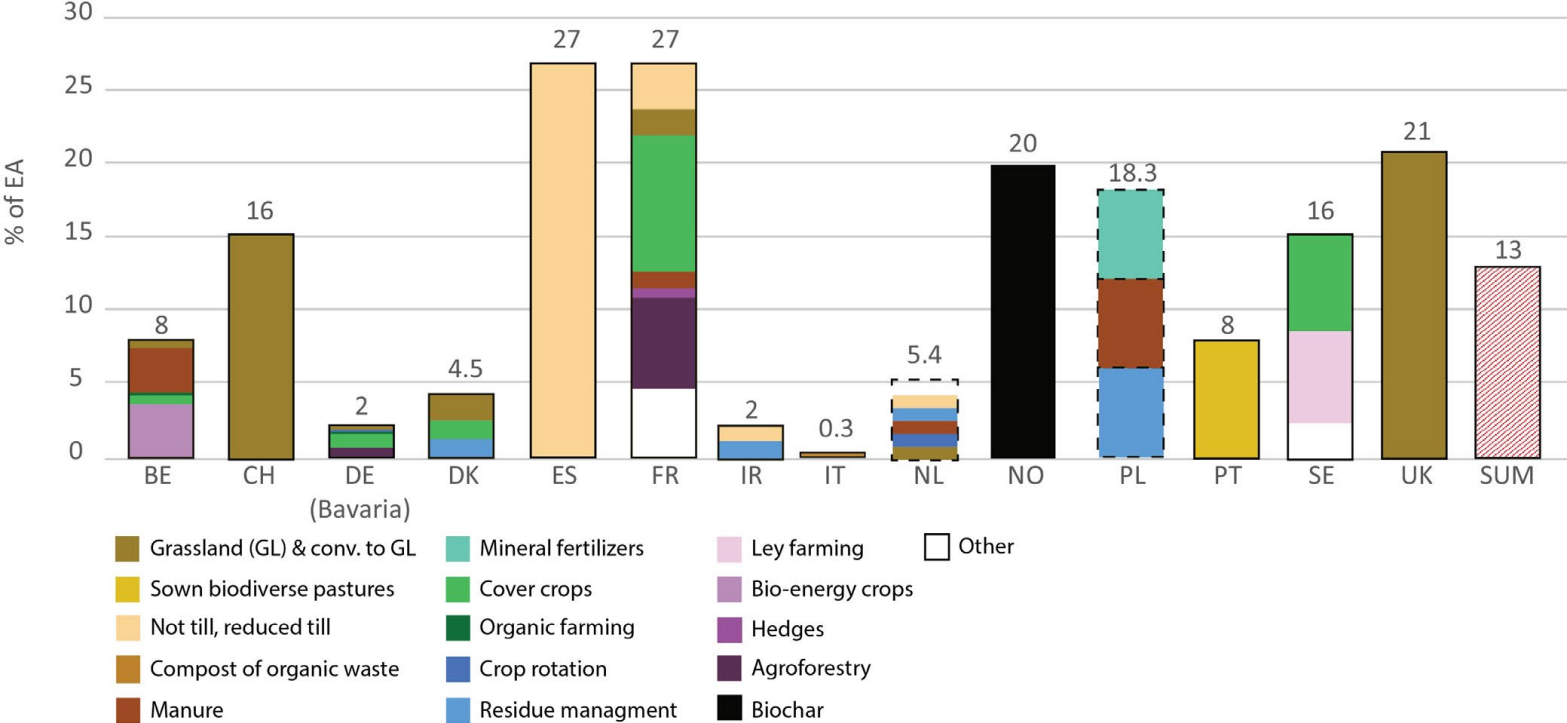
Shares of soil carbon sequestration potentials to offset the annual total emissions from the agricultural sector (EA) for each country and according to measures involved in the scenarios. Dashed squares indicate unknown shares of measures. Only measures with maximum potential per country are represented—additional potentials of countries are included in Table 2. Note that for France and Italy the conservative potentials are illustrated, which consider costs and respectively feasible compost production

Of the 24 countries participating in the study, about half of them could provide an estimate for SCS at national and regional scales at the time of the study. Achievable SCS in agricultural soils at the national scale was hence assessed for 13 countries (BE, CH, DK, ES, FR, IT, IE, NL, NO, PL, PT, SE and UK) while data for specific regions were available for three countries (BE, DE and IT). In total, 26 studies with either regional or national SCS estimates were considered. SCS potentials have been reported either as annual potentials (Tg C year^−1^ or Tg CO_2_ year^−1^) or as rates per unit of land (Mg C ha^−1^ year^−1^) and are summarized in Table 1. Further information was provided for the area of applicability and time period of the estimate. These numbers reflect current knowledge from partner countries, publication date, however, varies from 2003 to 2021 (median 2018), of which 30% are older than 5 years. Different countries applied contrasting approaches and methodologies and different combinations of measures for calculating SCS potentials (see Section 3.1 and 3.2). Estimates are reported for a certain soil depth, area and time period and therefore can hardly be compared directly to each other (Table 1). For this reason, the shares (%) of the potentials to the yearly domestic EA were calculated (Figure 1; Table 1).

**TABLE 1:** Achievable domestic soil carbon sequestration (SCS) reported for specific measures and temporal (years) and spatial scale

Country	Spatial Scale	Area (kha) of applied measure	Temp. Scale (years)	Measures	SCS‐Potential Tg C year^−1^	% EA ‰ of SOC stocks	Methods	Tier	References
Belgium	Regional (Flanders)	–	15	Permanent grassland, compost application, green manuring and management of crop residues (cereals)	0.05	–	Scenario analysis comparing change in an agricultural measure to ‘business as usual’ calculating SCS potential	Tier 2	D’Hose and Ruysschaert (2017; GL, Dutch)
	Regional (Flanders)	–	12	Green manuring, crop residue management, temporary pastures, organic farming and compost application	0.06	–	Increase of organic carbon in the ‘’current’’ (2002) and baseline (1990) years were calculated (sampling depth: 24 cm)	Tier 2	Sleutel et al. (2007; PSL, English)
	National	430.3	20	Bioenergy crops, farmyard manure, no‐tillage, cover crops, organic farming	0.214	8/1.17	Estimates are based on literature values and assumptions on the area the practice can be applied.	Tier 1	Dendoncker et al. (2004; GL, Dutch)
Denmark	National	2310	26	Cover crops, crops residue management, conversion to grassland	0.136	4.5/0.8	Modelling with C‐Tool (soil depth: 100 cm)	Tier 3	Taghizadeh‐Toosi and Olesen (2016; PSL, English)
France	National	28,500	30	Cover crops, reduced till, organic manures conversion to grassland, hedges, reduction of mowing, grass cover in vineyards, intra‐plot agroforestry	5.7 (30 cm) 2.9 (incl. costs)	28.5/2.2 14/1.1	Modelling (soil depth: 100 cm) with the STICS and PaSim models	Tier 3	Pellerin et al. (2019; GL, French)
	National		30	Cover crops, spatial insertion and temporal extension of temporary grasslands, improved recycling of organic resources as organic fertilizer	3	15/1.2	Modelling with STICS soil‐crop model (soil depth: 30 cm)	Tier 3	Launay et al. (2021; PSL, English)
Germany	Regional (Bavaria)	3315.8	–	Agroforestry, conversion to grassland, cover crops, improved crop rotation, organic farming	0.3–0.4	1.5–2.2/1.27	Estimate based on literature values and assumptions on the area the practices can be applied	Tier 1	Wiesmeier et al. (2020; PSL, English)
	Regional (Baden‐Württemberg)	3574.2	30	No‐tillage	0.285	1.6/2.8	Modelling with EPIC (Version 3060). Additional simulation of erosion by water and wind (100 cm)	Tier 2	Gaiser et al. (2008; PSL, English)
Ireland	National	160	10	Winter cover crops 0.51 Mg ha^−1^ year^−1^, winter cover crops combined with min. tillage 0.74 Mg ha^−1^ year^−1^	0.08–0.1	1.3–1.6/0.2	Flux measurements in combination with modelling and LTEs (soil depth: 15 cm)		Lanigan et al. (2012; GL)
	National	100	9	Reduced tillage (0.18–1 Mg ha^−1^ year^−1^), crops residue management (0.44–0.6 Mg ha^−1^ year^−1^)	0.11	2.2/0.21	Field experiments and modelling Roth and cohort model (soil depth: 60 cm)	Tier 3	van Groenigen et al. (2011; PSL)
	National	450	10	Improved management of grassland	0.07	1.3/0.14	Field experiments and modelling Roth and cohort model (soil depth: 60 cm)	Tier 3	Lanigan et al. (2018; GL)
Italy	National	16,284.1	100	Compost of organic waste	0.023	0.3/0.03	Modelling with RothC, 12 climate scenarios	Tier 3	Mondini et al. (2012; PSL, English)
	Regional (Apulia Region)	505.4	20	Compost of organic waste	0.031	–	Modelling with RothC10N and management scenarios	Tier 3	Bleuler et al. (2017; PSL, English)
	Regional (Apulia Region)	35.4	20	Crop residues incorporation and water management	0.002–0.02	–	Modelling with RothC10N and management scenarios	Tier 3	Di Bene et al. (2016; PLS, English)
Netherlands	National	–	20	Managing field margins, No‐tillage, non‐inversion till, green manure, crop residue management, reduction of grassland renovation, improved crop rotations	0.27	5.4/0.9	Modelling with MITERRA‐NL	Tier 2	Lesschen et al. (2012; GL Dutch)
	National	298.2	30	Improved crop rotation	0.245	5/0.8	Modelling with the program NDICEA version 6.2.1	Tier 2	Koopmans et al. (2019; GL Dutch)
	National	298.3	30	Reduction of grassland renovation	0.031	0.63/0.1	Modelling with the program NDICEA version 6.2.1	Tier 2	Koopmans et al. (2019; GL Dutch)
Norway	National	84	~100	Biochar	0.245	20/1.6	Based on meta‐analysis results by (Lehmann & Joseph, 2015)	Tier 1	Rasse et al. (2019; GL, Norwegian)
	National	1710	100	Cover crops	0.057	4.7/0.37	Estimate based on literature (Poeplau & Don, 2015).	Tier 1	Bøe et al. (2019; GL, Norwegian)
Poland	National	10,400	20	Reduced tillage, crops residues, manure	1.6	18/1.14	Modelling: DNDC model	Tier 3	Faber and Jarosz (2018; PSL, Polish)
Portugal	National	90	10	Sown biodiverse permanent pastures rich in legumes (SBPPR)	0.16	8/0.7	Modelling with a mass balance model for SOM dynamics (soil depth 10 cm)	Tier 2	Teixeira et al. (2011; PSL, English)
Spain	National	7650.6	10	No‐tillage	2.9	27/1.9	Modelling with the assessment tool CBP	Tier 2	Moreno‐García et al. (2020; PSL, English)
Sweden	National	1760	30	Perennials, intensification of leys, no bare fallowing, cover crops or catch crops	0.324	16/0.9	SOC change factors based on Swedish LTEs	Tier 2	Wikström (2019; GL, Swedish)
	National	600	20	Cover crops and agroforestry	0.144	7.3/0.4	SOC change factors based on Swedish long‐term trials. Land use projections are based on economic modelling.	Tier 2	Karlsson et al. (2020; GL, Swedish)
Switzerland	National	920	20	Grasslands	0.245 (max)	16/1.5	Estimate based on literature values and assumptions on the area these practices can be applied	Tier 1	Beuttler et al. (2019; GL, English)
	National	10	20	Deep ploughing	0.021	1.3/0.12	Estimate based on literature values and assumptions on the area these practices can be applied	Tier 1	Beuttler et al. (2019; GL, English)
	National	8.2	40	No‐tillage	0.0027	0.2/0.02	Estimate based on literature values and assumptions on the area these practices can be applied	Tier 1	Leifeld et al. (2003; GL, English)
UK	National	13,619	30	Grassland remaining grassland and cropland converted to grassland	2.39	21/1.6	UK Greenhouse Gas Inventory, 1990 to 2018	Tier 3	Brown et al. (2020; GL, English)

Data for the national carbon stocks from Lugato, Panagos, et al. (2014) except for Switzerland (Leifeld et al., 2005) and Bavaria in Germany (Wiesmeier et al., 2020) and Baden Württemberg (Poeplau et al., 2020). Last column also indicates the language of the source.

Abbreviations: EA, emissions from the agricultural sector %; ‰ of SOC stocks, share of the SCS potential to the national carbon stocks in ‰; PSL, published scientific literature; GL, grey literature; Tier, IPCC classification of the methodological approaches according level of complexity (1–3).

**FIGURE 2:**
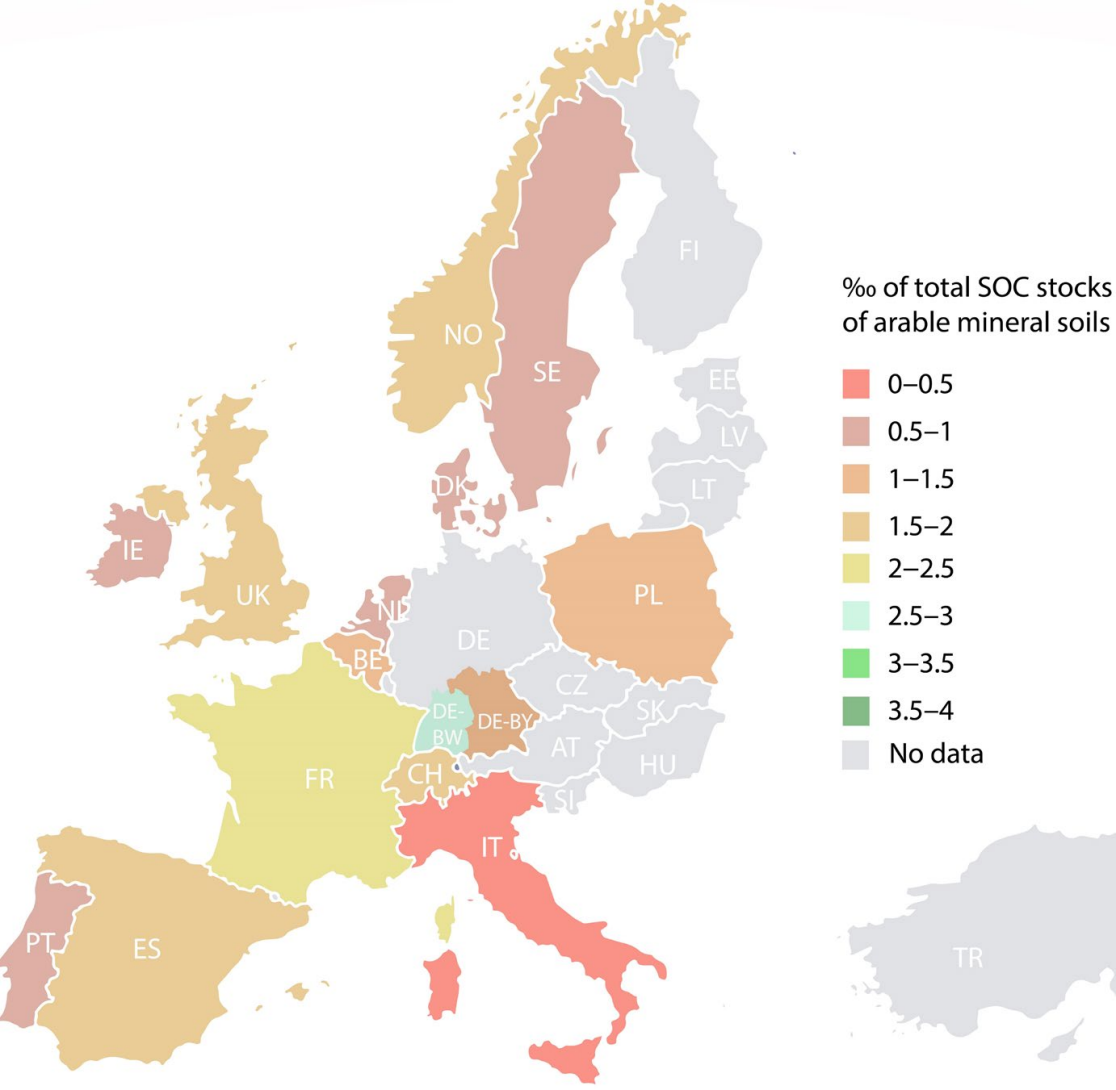
Feasibility of the 4 per 1000 initiative for the studied countries at national level. Colours represent the proportion of carbon in ‰ in relation to the national carbon stocks (2010) achieved with the highest national potential presented (see Table S2 for individual values). Note that potentials are not homogeneously distributed over the country but illustrate total estimated achievable potentials at national level

The total annual achievable SCS potential for climate change mitigation of each country varied greatly, ranging from 0.1% to 27% of EA (Table 1; Figure 1). The sum of all reported potentials (excluding repetitions of measures within one country; Table 1) totalled to 15.2 Tg C year^−1^. This would offset 13% of annual total European EA (Figure 1) for at least the next 9 years, which is the shortest time scale reported. In the context of the 4p1000 initiative, estimates were also converted into shares of current SOC stocks for agricultural land as reported by Lugato, Bampa, et al. (2014) (Figure 2). Annual SCS potentials ranged from 0.03‰ to 2.8‰ of the respective national soil carbon stocks, falling short of the 4p1000 target (Figure 2). The detailed description of country‐by‐country potentials including measures used is described in the supporting information.

### 3.1 Measures

**FIGURE 3:**
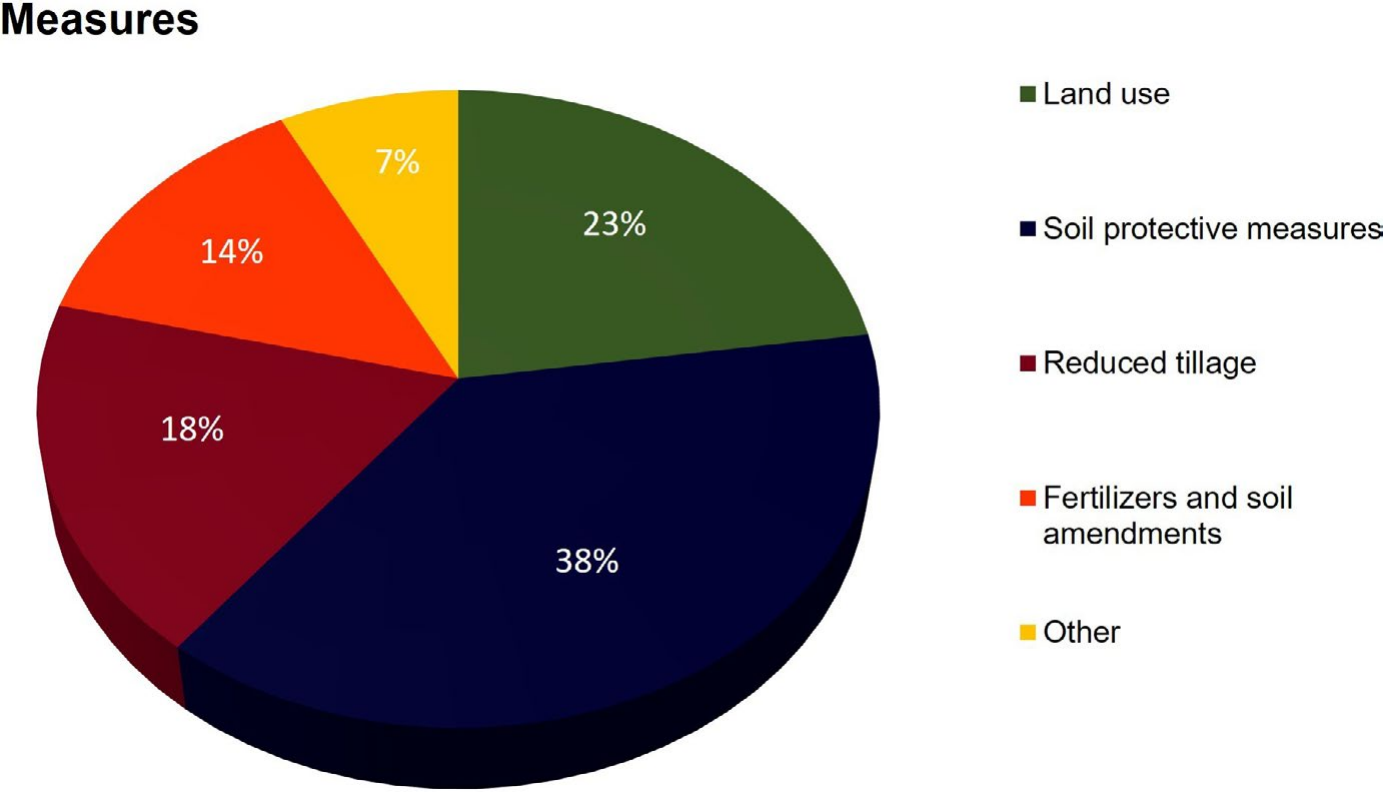
Share (%) of measures commonly identified and evaluated in national estimates of soil carbon sequestration

We evaluated the role of different measures in affecting national SCS potentials (Figures 1 and 3; Table 1). In total, 23 different measures were identified and studied to estimate SCS of mineral soils (see Table S1). Overall, 64 estimates of SCS potentials were identified in the available literature and related to different measures either alone or in combination. To analyse these results quantitatively, measures that were reported for SCS were classified into five main groups: (1) Land use change (excl. forest), (2) soil protection measures, (3) RT, (4) application of fertilizers and organic amendments and (5) other measures (Figure 3).

Under ‘land use change’, we included all measures that imply a long‐term change in the land cover, which cannot be quickly reversed (e.g. agroforestry [AGF]). Soil protective measures include all types of farming practices targeting or favouring soil protection such as CC, crop rotation and crop residue management. Reduced tillage practices, which generally fall into the soil protective category, were considered separately because of the broad diversity of RT practices. ‘Other measures’ contain all measures that do not fit into the other groups (see Table S1). Soil protective measures had the highest occurrence among studies, representing 38% of all reported measures (Figure 3). This group of measures includes CC (*n* = 9), crop residue management (RES; *n* = 7) and improved crop rotation (*n* = 2). Land use‐(change) measures accounted for 23% of all case studies and included land conversion to GR, *n* = 5 and AGF *n* = 3. RET measures occurred in with 18% of case studies. The three most mentioned single measures were CC (*n* = 9), RES (*n* = 7) and NT (*n* = 5). Total achievable SCS is commonly estimated by combining measures from all groups. Estimates of the achievable SCS using just one single measure were provided for NT (ES, DE and CH), cover crops (NO), perennial crops (SE) and biochar (NO).

### 3.2 Assessment of reported national achievable SCS

Contrasting methodologies were applied by the different countries for the technical assessment of SCS potentials, with variable levels of complexity regarding the inclusion or not of parameters such as trade‐offs in GHG emissions related to additional SCS, prediction of the impact of climate change or inclusion of socio‐economic parameters (i.e. costs, technical and practical feasibility).

#### 3.2.1 Technical assessment of reported SCS potentials

**FIGURE 4:**
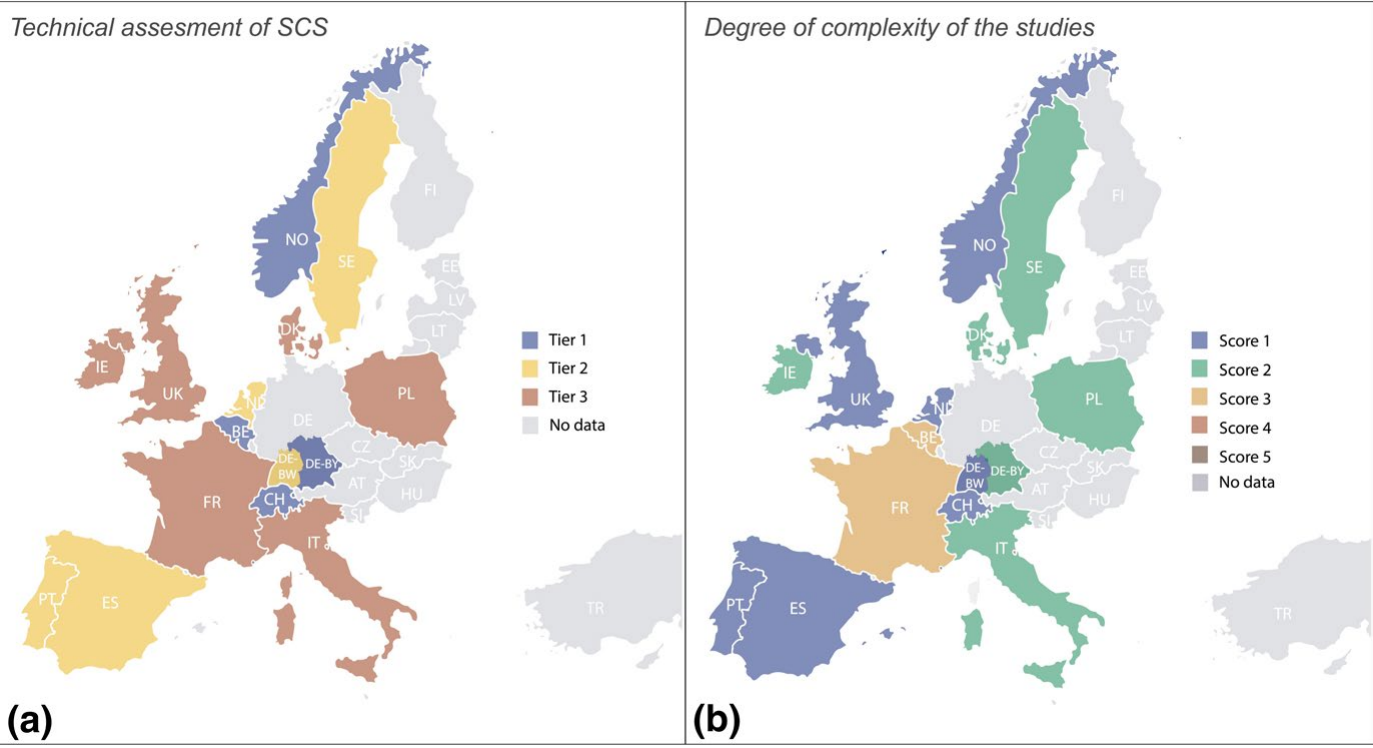
(a) Types of methodology (tiers 1 to 3) applied for MS countries for calculation of technical soil carbon sequestration (SCS) potential; and (b) degree of complexity of the national studies (Score 1–5) according to the number of controlling factors taken into account for the estimates (1. realistic areas, 2. technical and 3. practical feasibility, 4. trade‐offs with other GHG and 5. climate change). Note that potentials are not homogeneously distributed over the country but illustrate total estimated achievable potentials at national level

Three main approaches were used by the countries for estimating SCS: (1) SCS values included in published international literature studies (not country specific), (2) modelling outputs and (3) SOC change factors or flux measurements of GHG based on FE. For Norway, Switzerland and Belgium estimates of SCS rates were derived from literature associated with their agricultural areas. For Sweden SOC change estimates were provided by long‐term trials. In Flanders (BE) a comparison of soil C stocks was made between 1990 and 2002. Other countries including DK, DE (Baden Württemberg), ES, FR, IT, NL, PL and UK used a variety of modelling approaches (Table 1) to estimate SCS. The IPCC guidelines provide tiers (1–3) of standard methods for the estimation of SOC stocks and stock changes (IPCC, 2019). These tiers are characterized by both flexibility and complexity, ranging from Tier 1, which is based on default values provided by IPCC, to Tier 2 methods, which incorporate local information (country‐ or region‐specific data), to more advanced modelling and field‐based data included in the Tier 3 level. Figure 4a shows the tier level for each country where data are available and Table 1 provides an overview of the methods used for the estimation of SCS with distinct agricultural land use and management practises on mineral soils. Four countries (BE, CH, DE and NO) used a Tier 1 approach, five countries (ES, NL, PL, PT and SE) used a Tier 2 approach and six countries (DE (Baden Württemberg), DK, FR, IE, IT and UK) produced data according to a Tier 3 approach.

Different time periods were used to estimate SCS potentials; however, SCS does not increase linearly but tend to decrease over time. This means that SCS potentials, expressed as annual rates (Tg C year^−1^) will be larger if only calculated for a short time period (e.g. 10 or 20 years) and will decrease if calculated for a more extended time period (e.g. 100 years). The time periods considered in the EU studies ranged from 9 to 100 years and averaged 30 years. In total, seven studies reported time periods of less than 20 years. By definition, SCS related to a change in farming practices lasts only for a certain period of time, until soil carbon stocks reach a new steady state (except for biochar application which is more recalcitrant [Lehmann & Joseph, 2015]). The IPCC, therefore, recommends a time horizon of at least 20 years for estimating SCS potentials (IPCC, 2019).

Studies used different soil depths for their estimates, while IPCC recommends sampling to a minimum depth of 30 cm. The stocktake shows that 17% of the studies referred to soil depth <30 cm, 25% referred precisely to 30 cm, 37% considered soil >30 cm and 21% did not mention soil depth.

#### 3.2.2 Degree of complexity of the studies

For realistic estimates of achievable SCS, it is first essential to determine the land area where a particular measure can be implemented. Furthermore, costs and practical applicability of the measures should be considered. Climate change scenarios and trade‐offs with other GHG need also to be considered when estimating net SCS in the long term. No study has so far considered these parameters all together, and most studies simply provide a technical estimate of SCS potentials. The degree of complexity of the studies was evaluated here based on the number of parameters (Score 1 to 5) used within a particular study (Table 2 and Table S1 for each individual country), visualized in Figure 4b. More than half of the studies described in Table 1 used one single realistic parameter and the other 40% of the studies included between two and three parameters (Figure 4b). The highest score and thus most complete studies are given by Belgium (Dendoncker et al., 2004) and France (Pellerin et al., 2019) with Score 3 including a realistic area, technical and practical feasibility (Table S1; Figure 4b).

**TABLE 2:** Number of studies and countries using parameters (1–5) to estimate national SCS potential

Parameters	No. of studies	Countries
1. Realistic Area	5	BE, DE, DK, FR and SE
2. Technical feasibility	23	BE, CH, DE, DK, ES, FR, IT, IE, NL, NO, PL, PT, SE and UK
3. Practical feasibility	2	BE and FR
4. Climate Change	1	IT
5. Trade‐offs with N_2_O	2	IE and FR

##### Realistic area

The determination of the available area for implementation of a measure is mainly based on expert knowledge and available estimates from literature. Specific approaches for determination of the available area were applied by only four countries (BE, DE, DK and FR) (Table 2). These approaches include accounting for resource limitations (i.e. manure and straw availability) (Dendoncker et al., 2004; Pellerin et al., 2019; Taghizadeh‐Toosi & Olesen, 2016), national regulations that mandate specific measures or the use of fertilizers (Pellerin et al., 2019; Taghizadeh‐Toosi & Olesen, 2016) and specific soil properties and soil depths (i.e. non‐hydromorphic soils for NT) (Dendoncker et al., 2004; Pellerin et al., 2019). Concerning intra‐plot AGF, available plot size and soil depth seem to be important parameters for a realistic estimate of the implementation area (Pellerin et al., 2019). For the expansion of CC, the area occupied by winter crops and spring crops harvested too late to allow the sowing of a winter CC (e.g. potatoes, sugar beets and chicory), are taken into account for the state of Bavaria in Germany (Wiesmeier et al., 2020).

##### Technical and practical feasibility

According to our definition (see Section 2), most of the studies (*n* = 23) meet the technical feasibility. Measures which are still in the research stage, such as unproven technology or for which experiments are not yet locally available, do not meet a technical feasibility. Norway and Switzerland, for instance discuss the theoretical potential of incorporation of biochar into the soils (Beuttler et al. 2019; Rasse et al., 2019). Both studies recognize that biochar has a strong potential for SCS but also point out that the design of biochar application to soil has to primarily consider its effect on soil fertility, which is still poorly studied for temperate regions.

Practical feasibility is partly met by three countries (BE, IE and FR) which also take into account environmental and policy restrictions (BE; Dendoncker et al., 2004) and cost of measures and willingness of farmers to consider their implementation (FR; Pellerin et al., 2019). Finally only two countries (IE and FR) include the additional costs for farmers incurring when implementing measures. The study of Pellerin et al. (2019) for France shows that when acceptable costs are considered (e.g. 55 € t^−1^ CO_2_‐eq), the technical SCS potential of France is reduced by 50%. Nevertheless, some measures can also come with net savings such as for improved GR management in Ireland (IE; Lanigan et al., 2018), where costs for extra lime, clover seed, fuel and labour are overcompensated by the gain from higher grass yields.

##### Climate change and trade‐offs with other GHG (i.e. N_2_O)

Climate change is considered only in one study for Italy by Mondini et al. (2012). Changes in climate (temperature, precipitation and evapotranspiration) between 2001 and 2100 were predicted by 12 different scenarios, based on data from three different global circulation models (GCMs), namely HadCM3, PCM and GCM2 (Mitchell et al., 2004) and four different CO_2_ emission scenarios as defined in the IPCC Special Report on Emissions Scenarios (Nakicenovic et al., 2000). The study shows that SOC increase per unit area was negatively correlated with temperature. Therefore, the response to compost application in Italy was only 0.13 t C ha^−1^ year^−1^ when considering climate change. This rate is three times lower than values (0.4 ha^−1^ year^−1^) estimated by Smith et al. (2008) for manure and biosolids application and Freibauer et al. (2004) for compost amendment, where climate change was not accounted for (Mondini et al., 2012).

The results show that GHG emissions from soils and soil C sequestration are rarely measured simultaneously, even though they are both strongly affected by different management practices (Guenet et al., 2021). Two studies (FR and PL) include GHG emission other than CO_2_ to calculate net carbon emissions or removals. The Polish study of Faber and Jarosz (2018) presents simplified balances of carbon and gas absorption and emission of GHGs (CO_2_, CH_4_ and N_2_O) on regional levels over 20 years. Simulations were performed using the DNDC model for the different administrative units of Poland using a 20‐year series of meteorological data. The French study by Launay et al. (2021) takes into account several factors including GHG balance, biomass production and nitrogen‐ and water‐related impacts in addition to soil carbon stock changes. By using a high‐resolution modelling approach it was shown that current systems in France, even though they are accumulating some C in soils are on average strong sources of GHGs.

#### 3.2.3 Modelled technical and policy‐oriented scenarios versus bottom‐up national estimates

**FIGURE 5:**
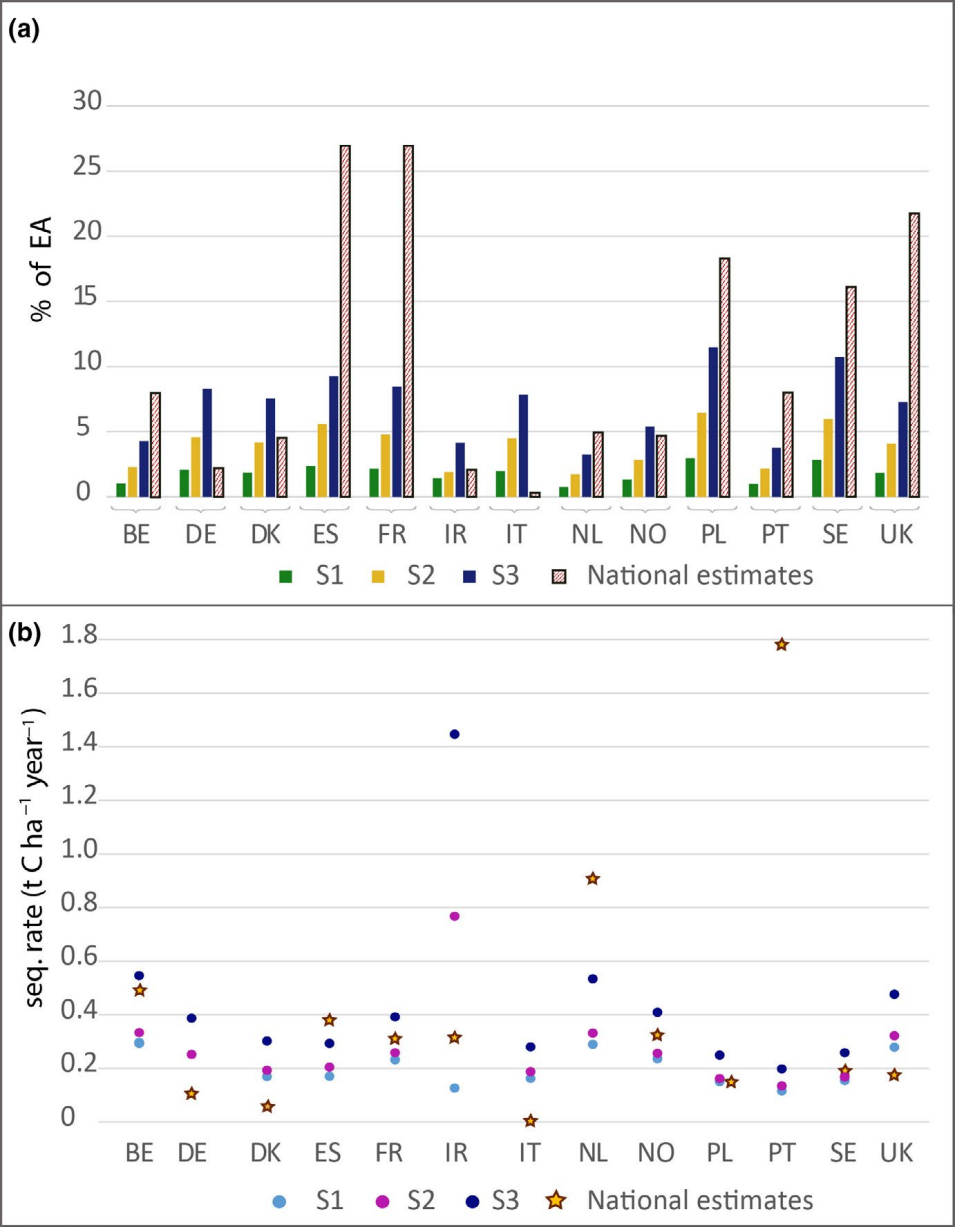
(a) Reduction potential (%) of greenhouse gas emissions from the agricultural sector for the three scenarios of Lugato, Bampa, et al. (2014) (named S1, S2 and S3) compared to the national estimates from this study. (b) Average soil sequestration (SCS) rates (t C ha^−1^ year^−1^) given by the three scenarios of Lugato, Bampa, et al. (2014) and the national estimates from this study

Panel (a) of Figure 5 shows the reduction potential of EA from the annual national estimates compared to the proposed policy maker scenarios by Lugato, Bampa, et al. (2014) calculated for each country (see Section 2). The potentials of the eight countries BE, ES, FR, NL, PL, PT, SE and UK are considerably higher than the modelled scenarios. If annual sequestration rates per unit land (ha) are considered, we get a slightly different picture, where SCS rates of BE, ES, NL and PT are equal or higher and the rest of the countries comprise lower SCS rates than the average rates given by the three scenarios (Figure 5b).

## 4 DISCUSSION

Estimates of achievable SCS across Europe remain highly uncertain mainly because a common SCS measuring and monitoring approach is not in place yet and because of the lack of data harmonization among countries in relation to the SCS potential of specific measures. Half of the partner countries included in this study lacked data on SCS at higher spatial levels (i.e. regional to national scale), while most of the remaining countries (BE, CH, DK, ES, FR, IR, IT, NL, NO, PO, SE and UK) provided nation‐wide achievable SCS potential estimates produced using different approaches. It is remarkable how data and country‐based knowledge on SCS potentials is highly heterogeneous regarding the choice of measures in each country, the methodological approach to assess SCS and the evaluation of other relevant parameters (e.g. economic and regulatory challenges) potentially affecting practical achievable SCS.

### 4.1 Management options for SCS across Europe

The most frequently suggested measures identified in previous studies for SCS at the European scale are CC, residue management (RES), conversion to permanent GR, reduced tillage (RET) and NT (Freibauer et al., 2004; Lugato, Bampa, et al., 2014; Vleeshouwers & Verhagen, 2002) but also the regular addition of animal manures to soils (Fornara et al., 2016; Freibauer et al., 2004; Smith et al., 1997). Based on national estimates the most important measures in decreasing order of frequency are CC, RES, RET, GR and NT. However, the selection of measures varies greatly among countries and while some countries focused on one single measure, others provided evidence of up to seven measures being applied to different proportions of land.

National estimates derived from the application of one single measure (CH, ES, NO, PT and UK) mainly reflected technical SCS potentials, while the countries using multiple measures (BE, FR and DK) included a higher degree of complexity of estimates (Figure 4b). Other less common measures such as AGF in France and bioenergy crops in Belgium appeared more important in terms of SCS potential than prominent measures like CC and permanent GR. This suggests that the potential to adopt realistic management practices also depends on current farms structure and resource availability. The study of Dendoncker et al. (2004), for instance, shows that the conversion of arable land to GR in Belgium is not likely to happen and that current GR area is rather decreasing. Taghizadeh‐Toosi and Olesen (2016) show that despite RES may increase SCS in Denmark, its potential is limited by the alternative fate of straw for fuel production, feed or bedding.

Several studies included RET and NT even though many European FE have shown that there is no or little effect on C stock changes when considering the whole soil profile (Anken et al., 2004; D’Hose et al., 2016; Dimassi et al., 2013; Feiziene et al., 2018; Hermle et al., 2008; Martínez et al., 2016; Meurer et al., 2018; Willekens et al., 2014). However, the effect of NT practices depends on pedo‐climatic conditions (Chenu et al., 2019; Kochiieru et al., 2020). NT and RET may be best suited for warm and dry conditions where positive effects on soil carbon stocks have been shown (Farina et al., 2018; López‐Bellido et al., 2020; Moreno‐García et al., 2020). NT, however, is associated with other agronomic benefits such as shorter periods with bare soil and better soil structure. This reduces soil erosion and increases water conservation, which may further justifies its application. This is particularly true for regions where water scarcity and erosion risks are limiting factors for agriculture (Álvaro‐Fuentes et al., 2014). Moreover, NT also allows longer periods for intercrops, which also promotes soil protection from erosion. Improving water availability and decreasing erosion risks might be a critical point in the context of climate change (Baveye et al., 2020), with the increasing occurrence of extreme climatic events such as prolonged drought and stormy rainfalls.

A highly debated measure currently adopted by some countries (BE, FR, NL and PL) for their SCS estimates is the application of animal manure. Manure is not always considered a SCS measure because it rather redistributes organic matter from one pool to another and is therefore not a net sink for CO_2_ (Chenu et al., 2019; Leifeld & Fuhrer, 2010; Schlesinger, 2000). However, Smith et al. (1997, 2000) state that using agricultural by‐products such as animal manure is crucial to recycling organic matter and to sequester carbon in soils. Also, the redistribution of manure previously applied on GR to arable land would lead to SCS as croplands have lower SOC contents (Dendoncker et al., 2004). In general, availability of manure is bound to animal husbandry, which has an intrinsically higher internal C return as compared to pure cash crop production where the share of exported biomass is much higher (Haberl et al., 2007).

Alternative measures like biochar and deep ploughing which are potentially interesting for many countries, were only mentioned by two countries (CH and NO). In recent years, biochar has been promoted as C negative emission technology and can also have positive effects on soil fertility and crop production (Lehmann, 2007; Smith, 2016). Biochar does not require extra additions of N fertilizer to sequester C in soils and thus can directly contribute to reducing N_2_O emissions (Guenet et al., 2021). However, biochar is a technology under development and questions of production costs and competition for feedstock with other bioenergy technologies need to be addressed. As biochar lasts in soil for centuries to millennia, a precautionary approach is indispensable and the in‐depth study of the conditions of biochar application to soil for a successful use, as well as an evaluation of associated risks, is urgently needed. Furthermore, biochars ability to influence the stabilization of plant‐derived C and other C inputs needs to be assessed across different pedo‐climatic conditions (Rasse et al., 2019).

Deep ploughing is a method used to improve soil structure and/or overcome hardpans of podzols, and could contribute to increase SOC stocks significantly (Alcántara et al., 2016; Schneider & Don, 2019). Nevertheless, it is essential to note that deep ploughing is an irreversible soil intervention influencing several soil ecosystem services (either positively or negatively), which needs to be assessed when optimizing soil fertility for crop production (Schiedung et al., 2019). Accordingly, the extent of the area where this specific measure is expected to be beneficial for soil fertility (podzols with hardpans) is relatively limited in European countries but might be significant at global scale.

### 4.2 Methodological approaches and assessment of SCS

The comparability of results among countries remains challenging due to the different methodological approaches implemented. There is, for example, a large variation in both soil depths and periods of time considered when estimating SCS. By definition, SCS following a change in farming practice will only last for a certain period until soil carbon stocks will reach a new steady state. The IPCC (2019) recommends a time horizon of at least 20 years to estimate SCS potentials. Findings from different LTEs, however, show how SCS can continue over many decades (Fornara et al., 2016; Poulton et al., 2018) and that soils may reach a new C equilibrium only after 100 years since a land‐use change (Johnston et al., 2017). Because rates of soil carbon accumulation are not linear and tend to decrease over time, annual rates of C accumulation (Tg C year^−1^) will be larger if calculated for a shorter period of time (e.g. 10 or 20 years) and will decrease if calculated for longer periods (e.g. 100 years). To harmonize methodologies and reduce variability, Smith et al. (2020) proposed a credible and reliable measurement, monitoring, reporting and verification platform, where countries can report and exchange approaches and data.

National achievable SCS potentials for different management options is still lacking mainly because of the complexity of measures, which involves transdisciplinary knowledge and the support of a variety of stakeholders. Available data from our study show that socio‐economic factors are rarely included in SCS potential studies. For instance, consideration of a realistic area where measures can be implemented, practical applicability and costs are generally not taken into account (Table 2). Socio‐economic factors, however, are key for a successful implementation of long‐term measures (Amundson & Biardeau, 2018; MacLeod et al., 2010). Improved information about the feasibility of implementing agricultural measures with high SCS potential, including the economic efficiency and social acceptability on a country scale, is needed. The study of Pellerin et al. (2019) for instance, shows that the implementation of most practices that sequester C will result in a cost to the farmers, whereas other measures are associated with essential co‐benefits (e.g. biodiversity, regulation of the water cycle, erosion reduction and other societal benefits), which are not yet monetized. Agroforestry, a relatively expensive measure for instance, comes with the simultaneous production of food and fibre, an increase in biodiversity, water and soil conservation, and improved resilience against climate change. Therefore, further efforts are needed to estimate the change in the value of co‐benefits as a result of soil management changes (Amelung et al., 2020; Pellerin et al., 2019). The valorization of co‐benefits could be key to meet social and economic acceptance. At the same time, it is also important that trade‐offs are considered.

There is an urgent need to consider the impact of climate change on agriculture to correctly design achievable SCS scenarios. At present, almost no studies include climate change scenarios for their estimates. Achievable C sequestration and GHG emissions from the agricultural sector must be considered in the context of climate change, which is expected to significantly affect, land use, production systems and farming practices in the near future. The study of Mondini et al. (2012) clearly shows the importance of including climate change scenarios and indicates that sequestration rates could be three times lower than expected because of climate change. Climate change will unevenly affect European regions and hence soil carbon dynamics (Kovats et al., 2014; Meersmans et al., 2016). Spatial predictions of future SOC stocks under different climate scenarios could be an important starting point for further investigation in critical regions with the highest SOC losses. However, there are still many uncertainties of how climate change affects the duration of vegetation periods, biomass and residue production and thus the effect of measures for SCS.

In terms of climate change mitigation, it is essential to include constant monitoring of GHG emissions, particularly N_2_O. Lugato et al. (2018) and Guenet et al. (2021) show that the GHG mitigation potential of soil carbon management can be significantly overestimated by neglecting N_2_O emissions that tend to increase with additional organic matter inputs. Only two studies (FR and PL) include GHG emission other than from CO_2_ to calculate net carbon emissions or removals. The study by Launay et al. (2021) highlights the importance of trade‐off (N_2_O) effects by showing that current systems in France, even though they are storing C in soils are, on average, strong GHG sources.

### 4.3 Comparing country‐specific estimates with the 4 per mille initiative

The aspirational goal set by the 4p1000 initiative is to increase SOC by 4‰ per year to a depth of 40 cm in all land uses. Our results suggest that this target under current management option is not feasible. All the estimated SCS potentials are, in general, low relative to the 4p1000 target ranging between 0.03‰ and 2.8‰ of the respective national or regional SOC stocks. Bruni et al. (2021) present similar findings for 14 European long‐term experimental sites and highlight the challenge of increasing SOC at a large scale especially under a warmer future climate.

However, the comparison of our data with the 4p1000 initiative has limitations: first, national SCS estimates reported are representative for different soil depths, while modelled soil carbon stocks refer to 30 cm soil depth (Lugato, Bampa, et al., 2014). The 4p1000 initiative on the other hand, refers to 40 cm. This difference in considered soil depth could lead to underestimations of SCS potentials. Second, modelled values are associated with uncertainties, as for example by poor model performance (e.g. underestimation of carbon stocks on coarse‐textured soils Ogle et al., 2007, see also Lugato, Panagos, et al., 2014). In addition, estimation of realistic areas of implementation is mostly missing and is probably overestimated.

### 4.4 Comparing country‐specific estimates to the modelled potentials

By comparing three policy‐oriented scenarios by Lugato, Bampa, et al. (2014) with the national estimates we find that most of national potentials are considerably higher than modelled potentials (BE, ES, FR, NL, PL, PT and UK). Results show that the sum of the reported potentials would amount to 15.2 Tg C year^−1^, which would offset 13% of the total European EA. This value is considerably higher than the previously suggested maximal annual potential of 9.1 Mt C (10 years; Lugato, Bampa, et al., 2014), which corresponds to 7.8% of the total European emissions from the agricultural sector. We find two possible reasons for the higher SCS estimates: One reason is that some countries (ES, PL and UK) assumed a considerably larger area of implementation. There is still high uncertainty concerning estimates of realistic areas. In some countries, ES and PL for instance, the estimated SCS potential would be reached only if projected measures were applied to the total agricultural area at national scale. This results in relatively high national potentials (Figure 5a), even though the sequestration rates t C ha^−1^ year^−1^ are similar or lower as the ones proposed by the scenarios of Lugato, Bampa, et al. (2014) (Figure 5b). The second reason is the higher average SCS rates achieved, which are mainly reached by those countries (BE, NL and PT) that suggest a combination of measures or measures specific to the country (Figure 5b). Sowing biodiverse pastures with legumes, for instance, a measure currently only applied in Portugal, shows relatively high potentials for the upper 10 cm of the soil (1.7 t C ha^−1^ year^−1^) (Teixeira et al.2011) (Figure 5b). The relatively high average SCS rate of Belgium is achieved by a combination of measures, of which bioenergy crops seems to play a key role with rates of 0.61+4.2 (fossil fuel savings) t C ha^−1^ year^−1^ applied on an area of 20 kha (Dendoncker et al., 2004).

Our study clearly shows that bottom‐up approaches and country‐specific expert knowledge are crucial to evaluate the achievable SCS and therefore, are complementary to homogeneous modelling approaches. The national potentials differ considerably from country to country and the considered measures go far beyond the most prominent measures (CC, GR, RES, RET and LEY) assessed by Lugato and others (Freibauer et al., 2004; Lugato, Bampa, et al., 2014; Vleeshouwers & Verhagen, 2002). The measures with the highest modelled technical potentials (conversion to GR) are, in most cases, limited to relatively small areas because of country‐specific farming situations.

### 4.5 Lessons for future SCS studies from available data on national estimates

Available country‐based knowledge on national achievable SCS potentials is still limited, and only half of the analysed partner countries have explored nationwide achievable SCS potential. Information provided often does not consider practical and socio‐economic implications, which are vital for sustainable implementation. The feasibility of the 4p1000 target seems highly questionable. However, national SCS potentials do suggest potentially important contributions to climate mitigation offsetting national GHG emissions from the agricultural sector in the range of 0.1% and 27% annually. Furthermore, national SCS estimated potentials presented here exceeded those from previous pan‐European modelling scenarios, underpinning the need for considering country‐ and region‐specific knowledge and expertise as a means for improvement and as complementary approach.

Comparisons among countries are limited by methodological heterogeneity. Although guidelines for technical assessment of C stock changes at various complexities (tiers) exist, a standard protocol to measure and compare achievable SCS is still missing. Even though many studies already use Tier 3 approaches, the degree of complexity of the studies, which is dependent on the five defined controlling factors is in general low. The degree of complexity of a study is, however, crucial for realistic and practical estimates of potentials. Efforts should be taken, not only to move towards higher tiers, but also to achieve higher degree of complexity in order to better inform policy makers and implement feasible and effective SCS measures. Future studies should also account for co‐benefits, when calculating costs. Many SCS measures are costly, and valorization of co‐benefits could be key for social and economic acceptance. Finally, the high heterogeneity of data urgently requests a harmonized approach to evaluate the achievable SCS.

## CONFLICT OF INTEREST

The authors declare that there is no conflict of interest.

## Supporting information

Supplementary Material

## Data Availability

The data that support the findings of this study are available in the supplementary material of this article.
