# Supplementary material for: Achievable agricultural soil carbon sequestration across Europe from country‐specific estimates
Source: Glob Chang Biol. 2021 Oct 6;27(24):6363–80. doi: 10.1111/gcb.15897 (PMC9293132; doi:10.1111/gcb.15897)
Supplement: Supplementary file 1 — Supplementary Material [file GCB-27-6363-s001.zip › gcb15897-sup-0001-Supinfo.docx]

# Supporting information

## Country by country description of potentials

**Belgium** would achieve a potential of 0.23 Tg C yr^-1^ (20 yrs.) on cropland, which could offset 8.5 % of national EA by combining measures including peatland restoration (Dendoncker et al., 2004). This national potential corresponds to 1.24 ‰ of the current soil carbons stock of Belgium (Fig. 2).

**Denmark** would reach a maximum potential of 0.136 Tg C yr^-1^ (26 yrs.) compensating 4.5 % of the EA by applying a combination of cover crops, crops residues management and conversion to grassland (Taghizadeh-Toosi and Olesen, 2016). This potential represents 0.8 ‰ of the Danish soil carbon stock (Fig. 2).

The potential 5.8 Tg C yr^-1^ (30 yrs.) of **France** corresponds to 28.5 % of EA and to 2.2 ‰ of the current carbon stocks, which states to be achieved by combining measures including agroforestry (Table 1, Fig. 1 and 2). However, this potential is associated with very high annual costs in the order of 2.3 billion euros. When acceptable costs are considered (e.g., 55 € t^-1^ CO_2_-eq), the potential is reduced by 50 % (Pellerin et al., 2019), which corresponds to 14 % of the EA and 1.1 ‰ of the French soil carbon stock (Fig. 1). Agroforestry, for instance, comes with relatively high costs, especially at the beginning because trees become profitable only after some years, presenting rather unfavourable policy incentives. The simultaneous production of food and fibre, an increase of biodiversity, water and soil conservation, and improved resilience against climate change are essential benefits.

A recent study by Launay et al. (2021) uses a high‐resolution modelling approach, which accounts for different GHGs from agricultural soils. The study shows that, by combining three different set of measures, a mean additional C storage of +184 (±179) kg C ha^-1^ yr^-1^ (30 yrs.) is achievable, which, , could mitigate up to 15 % of national yearly EA. These three set of measures include cover crops, spatial insertion and temporal extension of temporary grasslands, improved recycling of organic resources as organic fertilizer.

**Germany** presents estimates for two regions in SW and SE Germany. Comparing conventional and zero tillage and taking into account C losses through soil erosion the SCS potential for Baden Wüttemberg amounts 0.285 Tg C yr-1 (30 yrs.) (Gaiser et al., 2008). This would offset 1.6 % of the domestic EA of Baden Wüttemberg and represents 2.8 ‰ of the current soil carbon stock. Bavaria reports a potentials of 0.3-0.4 Tg C yr-1 for combining five measure including agroforestry (Wiesmeier et al., 2020), which would offset 1.5-2.2 % of the Bavarias EA and represent 1.3 ‰ of the current soil carbon stock.

**Ireland** reports maximal potential of 0.11 Tg C yr^-1^ (9 yrs.) for a combination of reduced tillage and crop residue management (van Groenigen et al., 2011). This would offset 2.2 % of the domestic EA and represents 0.21 ‰ of the current soil carbon stock.

The maximal potential SCS of 2.1 Tg C yr^-1^ (100 yrs.) reported for **Italy** could offset 25% of the EA by applying compost or organic waste to all the country's arable land. The application of compost at a national scale would improve the carbon stocks by 2.6 ‰. However, this maximal technical potential is reduced to 0.023 Tg C yr^-1^ when taking into account for the predictable and feasible compost production for 2020. Under these circumstances, the national reduction potential of GHGs from EA would be reduced by 0.3 %, corresponding to 0.03 ‰ of the current carbon stocks. The study, however, identifies areas with the greatest potential for the accumulation of organic matter. This information could be valuable for policymakers and help them to put the focus where it matters the most (Mondini et al., 2012).

The maximal potential reachable by **the** **Netherlands,** combining six different measures (see table 1), amounts in 0.27 Tg C yr^-1^ (20 yrs.) (Lesschen et al., 2012), which would potentially abates 5.4 % of the EA and correspond to 0.9 ‰ of the national carbon stocks. Further potentials are given by Koopmans et al. (2019) for improved crop rotation (0.25 Tg C yr^-1^ [30 yrs.]) and reduction of grassland renovation (0.06 Tg C yr^-1^ [30 yrs.]), offsetting GHG from EA by 5 % and 0.63 % and improving carbon stocks by 0.8 and 0.1 ‰, respectively.

**Norway** reports soil biochar application as the measure with the most outstanding potential of 0.245 Tg C yr^-1^ (~100 yrs.) for storing SOC in Norwegian soils on a long-term perspective, which would offset 20 % of their GHG emissions from the agricultural sector and would correspond to 1.6 ‰ of the national carbon stocks. However, it has to be noted that the production of biochar would rely on 50 % of the available biomass residues at the national scale and would therefore compete with other uses for this feedstock (Rasse et al., 2019). As biochar remains mostly stable in the soil, the timeframe during which the method can be used to increase soil C exceeds 100 years. However, there is a timeframe of at least a decade for deploying a technology that only exist at the pilot stage for now.

An increased use of cover crops during the intercropping period up to 60 % of the total cropland area would amount to 0.057 Tg C yr^-1^ (50 yrs.), offsetting 4.7 % of the EA. Today only 0,8 % of the total cropland area is covered during intercropping, hence current SCS corresponds to a reduction of 0.0070 Tg C yr^-1^ (Bøe et al., 2019).

For **Poland**, a SCS potential of 1.6 Tg C yr^-1^ (20 yrs.) was calculated for the optimized use of reduced tillage, crops residues and manure application. This corresponds to 18 % of EA It should be noted that this potential is only accomplishable if the total cropland area of Poland is considered (Faber and Jarosz, 2018). This potential represents 1.14 ‰ of the Polish soil carbon stocks.

A potential of 0.16 Tg C yr^-1^ (10 yrs.) compensating 8 % of national EA could be achieved by **Portugal** by means of the so called *sown biodiverse permanent pastures rich in legumes* (SBPPRL), a specific grassland system (Teixeira et al., 2011). This additional SCS potential corresponds to 0.7 ‰ of the national soil carbons stocks. Even though Portugal is the only country using this measure, its application might also have great potential on other areas with Mediterranean characteristics.

For **Spain,**a SCS potential of 2.9 Tg C yr^-1^ (10 yrs.), corresponding to 1.9 ‰ of the soil carbon stock and to 27 % of their EA, would be attained by implementing no-till to the total cropland area of Spain. The relative importance of additional SCS expected from no tillage according to local conditions was assessed within a regional framework and identifies priority regions for its implementation (Moreno-García et al., 2020).

A potential SCS of 0.324 Tg C y^-1^, is given by **Sweden** compensating 16 % of domestic EA could be achieved by applying a combination of perennials (ley), permanent grassland, cover crops or catch crops (Wikström, 2019). This represents 0.9 ‰ of Sweden’s total soil carbon stocks. A further potential SCS of 0.14 Tg C yr^-1^ (20 yrs.) is reached by combining cover crops and agroforestry. This potential would offset 7.3% % of the national EA  (Karlsson et al., 2020) and represent 0.39 ‰ of the total soil carbon stocks.

**Switzerland** and **UK** report a maximum technical potential for extension of permanent grassland area with 0.245 Tg C yr^-1^ (Beuttler et al., 2019) and 2.39 Tg C yr^-1^ (Brown et al., 2020), respectively. This would account for 16 % and 21 % of their respective EA. For Switzerland, this would correspond to 1.5 ‰ of current SOC stocks and 1.6 ‰ for UK. Preliminary estimates made for Switzerland show that applying deep ploughing on an area of 5000 ha per year could offset the emissions of 15.4 million t CO2 over 20 years (Beuttler et al., 2019). This would compensate for 12 % of the annual emission from the agricultural sector.

Table S1 Agricultural measures to improve SCS in mineral soils mentioned by the studies analysed.

| **Measures** | **Total number of studies** |
| --- | --- |
|  |  |
| **1 Land use:** | **15** |
| Permanent grassland | 6 |
| Conversion to grassland | 5 |
| Sown biodiverse permanent grasslands | 1 |
| Agroforestry | 3 |
| **2 Soil protective measures:** | **25** |
| Ley farming | 2 |
| Cover crops | 9 |
| Catch crop | 2 |
| Improved crop rotation | 2 |
| Crop residue management | 7 |
| Reduction of grassland renovation | 3 |
| **3 Tillage:** | 12 |
| No-till | 5 |
| Conservation tillage (minimum tillage, reduced tillage) | 5 |
| Non inversion tillage | 1 |
| Deep ploughing | 1 |
| **4 Fertilizers and amendments:** | 9 |
| Mineral fertilizers | 2 |
| Manure application | 2 |
| **Amendments:** |  |
| Compost of organic waste | 3 |
| Biochar | 2 |
| **5 Other measures:** | 5 |
| Managed field margins | 2 |
| Organic farming | 2 |
| Energy crops | 1 |

Table S2 Degree of complexity of studies defined by six controlling factors.

| **MS** | **Realistic Area** | **Technical feasibility** | **Practical feasibility** | **Climate change** | **Other GHG** | **Score** | **Reference** |
| --- | --- | --- | --- | --- | --- | --- | --- |
| BE | + | + | + | - | - | 3 | Dendoncker et al., 2004 |
| BE | - | + | - | - | - | 1 | Sleutel et al., 2007 |
| BE | - | + | - | - | - | 1 | D’Hose & Ruysschaert 2017 |
| CH | - | + | - | - | - | 1 | Beuttler et al., 2019 |
| CH | - | - | - | - | - | 1 | Leifeld et al., 2003 |
| DE | + | + | - | - | - | 2 | Wiesmeier et al., 2020 |
| DE | - | + | - | - | - | 1 | Gaiser et al., 2008 |
| DK | + | + | - | - | - | 2 | Taghizadeh-Toosi and Olesen, 2016 |
| ES | - | + | - | - | - | 1 | Moreno-García et al., 2020 |
| FR | + | + | + | - | - | 3 | Pellerin et al., 2019 |
| FR | - | + | - | - | + | 2 | Launay et al.2021 |
| IR | - | + | - | - | + | 2 | Lanigan et al 2012 |
| IR | - | + | - | - | + | 2 | Lanigan et al 2018 |
| IR | - | + | - | - | - | 1 | van Groenigen et al., 2011 |
| IT | - | + | - | + | - | 2 | Mondini et al., 2012 |
| IT | - | + | - | - | - | 1 | Bleuler et al., 2017 |
| IT | - | + | - | - | - | 1 | Di Bene et al., 2016 |
| NL | - | + | - | - | - | 1 | Lesschen et al., 2012 |
| NL | - | + | - | - | - | 1 | Koopmans et al., 2019 |
| NO | - | - | - | - | - | 0 | Rasse et al., 2019 |
| NO | - | + | - | - | - | 1 | Bøe et al., 2019 |
| PO | - | + | - | - | + | 2 | Faber and Jarosz, 2018 |
| PT | - | + | - | - | - | 1 | Teixeira et al., 2011 |
| SE | - | + | - | - | - | 1 | Wikström, 2019 |
| SE | + | + | - | - | - | 2 | Karlsson et al., 2020 |
| UK | - | + | - | - | - | 1 | Brown et al., 2020 |

Table S3 Summery of SCS potentials in realtion to emissions from the agricultural sector and Carbon stocks and –changes.

| **MS** | **SCS-Potential** | **Compensation of EA (%)** | **EA** | **SOC stock by Lugato et al. 2014a** | **∆ SOC** | **Reference** |
| --- | --- | --- | --- | --- | --- | --- |
|  | **Tg C/yr** | **(%)** | **(Tg C)** | **Tg C** | **‰** |  |
| BE | 0.214 | 2.2 | 2.698 | 183.245 | 1.17 | Dendoncker et al., 2004 |
| CH | 0.258 | 15.8 | 1.632 | 170* | 1.5 | Beuttler et al., 2019 |
| CH | 0.0027 | 0.2 | 1.632 | 170* | 0.02 | Leifeld et al., 2003 |
| DE | 0.4 | 2.2 | 18.3 | 267** | 1.27 | Wiesmeier et al., 2020 |
| DE | 0.285 | 1.6 | 18.3 | 101.0*** | 2.82 | Gaiser et al., 2008 |
| DK | 0.136 | 4.5 | 3 | 178 | 0.76 | Taghizadeh-Toosi and Olesen, 2016 |
| ES | 2.9 | 27 | 10.7 | 1539.84 | 1.9 | Moreno-García et al., 2020 |
| FR | 5.7 | 27 | 20.6 | 2566.7 | 2.22 | Pellerin et al., 2019 |
| FR | 3 | 14.5 | 20.6 | 2566.7 | 1.27 | Launay et al.2021 |
| IR | 0.08 – 0.1 | 1.29 – 1.6 | 5.2 | 518 | 0.15-0.19 | Lanigan et al 2012 |
| IR | 0.07 | 1.3 | 5.2 | 518 | 0.14 | Lanigan et al 2018 |
| IR | 0.11 | 2.1 | 5.2 | 518 | 0.19 | van Groenigen et al., 2011 |
| IT | 0.023 | 0.3 | 8.2 | 810 | 0.03 | Mondini et al., 2012 |
| NL | 0.27 | 5.4 | 4.96 | 292.73 | 0.92 | Lesschen et al., 2012 |
| NL | 0.245 | 4.9 | 4.96 | 292.73 | 0.84 | Koopmans et al., 2019 |
| NO | 0.245 | 20.1 | 1.22 | 154.75 | 1.58 | Rasse et al., 2019 |
| NO | 0.057 | 4.7 | 1.22 | 154.75 | 0.37 | Bøe et al., 2019 |
| PO | 1.58 | 18.3 | 8.6 | 1389.76 | 1.14 | Faber and Jarosz, 2018 |
| PT | 0.16 | 8.04 | 1.99 | 232.3 | 0.69 | Teixeira et al., 2011 |
| SE | 0.144 | 7.3 | 1.96 | 372.39 | 1.24 | Karlsson et al., 2020 |
| SE | 0.324 | 16.5 | 1.96 | 372.39 | 0.87 | Wikström et al., 2019 |
| UK | 2.39 | 21.4 | 11.23 | 1466 | 1.63 | Brown et al., 2020 |

* (Leifeld et al., 2005)

** (Wiesmeier et al., 2020)

*** (Poeplau et al., 2020)

Bøe, F., Bechmann, M., Øgaard, A.F., Sturite, I., Brandsæter, L.O., 2019. Fangvekstenes økosystemtjenester – Kunnskapsstatus om effekten av fangvekster (Nibio No. 5). Norway.

Brown, P., Cardenas, L., Choudrie, S., Jones, L., Karagianni, E., MacCarthy, J., Passant, N., Richmond, B., Smith, H., 2020. UK Greenhouse Gas Inventory, 1990 to 2018: Annual Report for submission under the Framework Convention on Climate Change - Defra, UK. Department for Environment, Food and Rural Affairs (Defra), Nobel House, 17 Smith Square, London SW1P 3JR helpline@defra.gsi.gov.uk.

Dendoncker, N., Van Wesemael, B., Rounsevell, M.D.A., Roelandt, C., Lettens, S., 2004. Belgium’s CO2 mitigation potential under improved cropland management. Agric. Ecosyst. Environ. 103, 101–116. https://doi.org/10.1016/j.agee.2003.10.010

Faber, A., Jarosz, Z., 2018. Modelowanie emisji podtlenku azotu i amoniaku w skali regionalnej oraz w Polsce. Zesz. Nauk. SGGW W Warszawie - Probl. Rol. Świat. 18(33), 70–81. https://doi.org/10.22630/PRS.2018.18.2.35

Gaiser, T., Stahr, K., Billen, N., Mohammad, M.A.-R., 2008. Modeling carbon sequestration under zero tillage at the regional scale. I. The effect of soil erosion. Ecol. Model. 218, 110–120.

Karlsson, A.-B., Boström, B., Jernbäcker, E., Lundblad, M., Mjureke, D., 2020. Vägen till en klimatpositiv framtid. SOU 2020: Betänkande från Klimatpolitiska vägvalsutredningen (M 2018:07).

Koopmans, C., Timmermans, B., Wagenaar, J.P., van, J., 2019. Evaluatie van maatregelen voor het vastleggen van koolstof 54.

Launay, C., Constantin, J., Chlebowski, F., Houot, S., Graux, A., Klumpp, K., Martin, R., Mary, B., Pellerin, S., Therond, O., 2021. Estimating the carbon storage potential and greenhouse gas emissions of French arable cropland using high‐resolution modeling. Glob. Change Biol. 27, 1645–1661. https://doi.org/10.1111/gcb.15512

Leifeld, J., Bassin, S., Fuhrer, J., 2005. Carbon stocks in Swiss agricultural soils predicted by land-use, soil characteristics, and altitude. Agric. Ecosyst. Environ. 105, 255–266. https://doi.org/10.1016/j.agee.2004.03.006

Lesschen, J.P., Heesmans, H.I.M., Mol-Dijkstra, J.P., Doorn, A.M. van, Verkaik, E., Wyngaert, I.J.J. van den, Kuikman, P.J., 2012. Mogelijkheden voor koolstofvastlegging in de Nederlandse landbouw en natuur. Alterra.

Mondini, C., Coleman, K., Whitmore, A.P., 2012. Spatially explicit modelling of changes in soil organic C in agricultural soils in Italy, 2001–2100: Potential for compost amendment. Agric. Ecosyst. Environ. 153, 24–32. https://doi.org/10.1016/j.agee.2012.02.020

Moreno-García, M., Repullo-Ruibérriz de Torres, M.Á., González-Sánchez, E.J., Ordóñez-Fernández, R., Veroz-González, Ó., Carbonell-Bojollo, R.M., 2020. Methodology for estimating the impact of no tillage on the 4perMille initiative: The case of annual crops in Spain. Geoderma 371, 114381. https://doi.org/10.1016/j.geoderma.2020.114381

Pellerin, S., Bamière, L., Launay, C., Martin, R., Schiavo, M., Angers, D., Augusto, L., Balesdent, J., Doelsch, I.B., Bellassen, V., 2019. Stocker du carbone dans les sols français, quel potentiel au regard de l’objectif 4 pour 1000 et à quel coût?

Poeplau, C., Jacobs, A., Don, A., Vos, C., Schneider, F., Wittnebel, M., Tiemeyer, B., Heidkamp, A., Prietz, R., Flessa, H., 2020. Stocks of organic carbon in German agricultural soils—Key results of the first comprehensive inventory. J. Plant Nutr. Soil Sci. 183, 665–681. https://doi.org/10.1002/jpln.202000113

Rasse, D., Økland, I.H., Bárcena, T.G., Riley, H., Martinsen, V., Sturite, I., Joner, E., O’Toole, A., Øpstad, S., Cottis, T., 2019. Muligheter og utfordringer for økt karbonbinding i jordbruksjord. NIBIO Rapp.

Taghizadeh-Toosi, A., Olesen, J.E., 2016. Modelling soil organic carbon in Danish agricultural soils suggests low potential for future carbon sequestration. Agric. Syst. 145, 83–89. https://doi.org/10.1016/j.agsy.2016.03.004

Teixeira, R.F.M., Domingos, T., Costa, A., Oliveira, R., Farropas, L., Calouro, F., Barradas, A.M., Carneiro, J., 2011. Soil organic matter dynamics in Portuguese natural and sown rainfed grasslands. Ecol. Model. 222, 993–1001.

van Groenigen, K.J., Hastings, A., Forristal, D., Roth, B., Jones, M., Smith, P., 2011. Soil C storage as affected by tillage and straw management: An assessment using field measurements and model predictions. Agric. Ecosyst. Environ. 140, 218–225. https://doi.org/10.1016/j.agee.2010.12.008

Wiesmeier, M., Mayer, S., Burmeister, J., Hübner, R., Kögel-Knabner, I., 2020. Feasibility of the 4 per 1000 initiative in Bavaria: A reality check of agricultural soil management and carbon sequestration scenarios. Geoderma 369, 114333. https://doi.org/10.1016/j.geoderma.2020.114333

Wikström, L., 2019. Så klarar det svenska jordbruket klimatmålen: en delrapport från IVA-projektet Vägval för klimatet.

Stockholm, ISBN: 978-91-7082-990-1.
